# Using mobile technology to engage sexual and gender minorities in clinical research

**DOI:** 10.1371/journal.pone.0216282

**Published:** 2019-05-02

**Authors:** Mitchell R. Lunn, Matthew R. Capriotti, Annesa Flentje, Kirsten Bibbins-Domingo, Mark J. Pletcher, Antony J. Triano, Chollada Sooksaman, Jeffrey Frazier, Juno Obedin-Maliver

**Affiliations:** 1 The PRIDE Study/PRIDEnet, University of California, San Francisco, San Francisco, California, United States of America; 2 Division of Nephrology, Department of Medicine, School of Medicine, University of California, San Francisco, San Francisco, California, United States of America; 3 UCSF Center for Vulnerable Populations, San Francisco, California, United States of America; 4 Department of Psychology, San Jose State University, San Jose, California, United States of America; 5 Department of Community Health Systems, School of Nursing, University of California, San Francisco, San Francisco, California, United States of America; 6 Department of Epidemiology and Biostatistics, School of Medicine, University of California, San Francisco, San Francisco, California, United States of America; 7 Division of General Internal Medicine, Department of Medicine, School of Medicine, University of California, San Francisco, San Francisco, California, United States of America; 8 THREAD Research, Tustin, California, United States of America; 9 Department of Obstetrics, Gynecology & Reproductive Sciences, School of Medicine, University of California, San Francisco, San Francisco, California, United States of America; USC Keck School of Medicine, Institute for Global Health, UNITED STATES

## Abstract

**Introduction:**

Historical and current stigmatizing and discriminatory experiences drive sexual and gender minority (SGM) people away from health care and clinical research. Being medically underserved, they face numerous disparities that make them vulnerable to poor health outcomes. Effective methods to engage and recruit SGM people into clinical research studies are needed.

**Objectives:**

To promote health equity and understand SGM health needs, we sought to design an online, national, longitudinal cohort study entitled The PRIDE (Population Research in Identity and Disparities for Equality) Study that enabled SGM people to safely participate, provide demographic and health data, and generate SGM health-related research ideas.

**Methods:**

We developed an iPhone mobile application (“app”) to engage and recruit SGM people to The PRIDE Study–Phase 1. Participants completed demographic and health surveys and joined in asynchronous discussions about SGM health-related topics important to them for future study.

**Results:**

The PRIDE Study–Phase 1 consented 18,099 participants. Of them, 16,394 provided data. More than 98% identified as a sexual minority, and more than 15% identified as a gender minority. The sample was diverse in terms of sexual orientation, gender identity, age, race, ethnicity, geographic location, education, and individual income. Participants completed 24,022 surveys, provided 3,544 health topics important to them, and cast 60,522 votes indicating their opinion of a particular health topic.

**Conclusions:**

We developed an iPhone app that recruited SGM adults and collected demographic and health data for a new national online cohort study. Digital engagement features empowered participants to become committed stakeholders in the research development process. We believe this is the first time that a mobile app has been used to specifically engage and recruit large numbers of an underrepresented population for clinical research. Similar approaches may be successful, convenient, and cost-effective at engaging and recruiting other vulnerable populations into clinical research studies.

## Introduction

Understanding the health needs of vulnerable populations–and the inequities that contribute to those needs–is critical to improving health and wellbeing. Engaging vulnerable populations, however, is often challenging because these populations are underserved in clinical settings, understudied in research, and experience structural and societal barriers to wellness including institutionalized discrimination and stigmatized identities. Unethical conduct from investigational communities (*e*.*g*., Tuskegee syphilis experiments on African-Americans, harmful conversion “therapies” to change sexual orientations and gender identities, limited initial federal response to the HIV epidemic) has broken the trust of many populations and deterred them from interacting with the health care system. Some populations–such as sexual and gender minority (SGM) people–are particularly difficult to identify or find because their demographic information (*e*.*g*., sexual orientation, gender identity) is not captured by health care systems [1], reported in clinical research [2, 3], or included in federal population surveys (*e*.*g*., Census, American Community Survey) which are used to generate probability-based sampling frames for larger national health studies [4]. These absent data make SGM people invisible [5, 6].

Digital engagement for clinical research may be an effective strategy with vulnerable populations for five reasons. First, digital interactions (*i*.*e*., with a website or a mobile app) can be free of stigmatizing or discriminatory interpersonal interactions (*e*.*g*., homophobia, transphobia) [7–10]. Second, the ubiquity of Internet access via numerous devices (*e*.*g*., computers, tablets, smartphones) enables a broad reach into geographic areas away from traditional research centers [11, 12]. Third, customization enables unique experiences composed only of study components applicable to a specific participant (*e*.*g*., tailored survey language and questions based on demographics or reported behaviors). This also permits study participants to consent to sharing specific aspects of their participation and designating who can receive those data [13]. Fourth, the participant remains in full control of their participation and can easily determine the specific study components they would like to perform without the pressure of an in-person interaction during which they may feel obligated to maintain participation or embarrassed to refuse specific study components. Finally, digital approaches are nimble and can be rapidly modified as needs dictate including due to societal changes and in response to participant feedback. Despite these advantages, digital engagement strategies do have pitfalls and may be unsuccessful for several reasons. First, they do not allow meaningful in-person interpersonal interactions and relationship-building, which may help foster engagement and retention in research studies. Second, verifying participant identity is difficult and expensive in a digital-only setting. Third, digital interactions require less participant effort than in-person engagement; while too much effort will deter participation, too little effort may result in non-committed participants who are subject to drop-out and loss to follow-up. Finally, while the vast majority has Internet access, Internet access may be limited for those older than 65 years, with less annual income, with less education, and in rural areas [14].

The near-ubiquity of Internet access (89% of US adults [14] and increasing smartphone ownership (77% of US adults [15]) have opened new avenues to influence and study health in participants’ natural environments using tools (*i*.*e*., smartphone, Internet access) already in participants’ possession. Health-related mobile applications (“apps” [16]) are commonly available for behavior tracking (*e*.*g*., tracking diet, weight, exercise), often in conjunction with a connected consumer device (*e*.*g*., physical activity tracker, scale). Additional health-related apps provide coaching (*e*.*g*., weight loss, smoking cessation) or facilitate interactions with the health care system by connecting to patient portals in electronic health records. While significantly fewer apps exist for the express purpose of conducting health-related research, the recent advent of the open-source software development frameworks ResearchKit (for iOS, researchkit.org) and ResearchStack (for Android, researchstack.org) enabled rapid app development and fostered researcher-software developer partnerships to study a variety of conditions from heart disease [17] to asthma [18, 19] and from melanoma [20] to Parkinson disease [21]. This paradigm shift in clinical research simultaneously pushed novel methods to facilitate scalable participant-administered consent processes [22].

Sexual and gender minority (SGM) people (*i*.*e*., lesbian, gay, bisexual, transgender, and queer communities and people whose sexual orientation, gender identity/expression, or reproductive development varies from traditional, societal, cultural, or physiological norms [23]) are an underserved, vulnerable, and understudied population [24]. To promote SGM health equity and understand the health needs of SGM people, we designed an online, national, longitudinal cohort study entitled The PRIDE (Population Research in Identity and Disparities for Equality) Study (pridestudy.org). Because SGM people have been subject to stigma and discrimination in society and by the medical and investigational communities [25, 26], we used a digital approach to promote safe, comfortable participation by SGM people. We desired to partner with SGM community members to learn about them and their health concerns and to actively engage them in designing The PRIDE Study.

Here, we report the development of a novel ResearchKit-based iPhone app for The PRIDE Study–Phase 1. We used the app for three main goals: (i) to recruit a large sample of diverse SGM people [27], (ii) to collect demographic and health data, and (iii) to engage SGM people in the generation of SGM health-related research ideas. This paper focuses on the development of our iPhone app, the successful recruitment of diverse SGM people, and their engagement with study activities (surveys, community forum). (Health survey data and participant-proposed SGM health research topics will be reported elsewhere.) We believe this is the first time that a mobile app has been used to specifically recruit and engage large numbers of an underrepresented and understudied population for clinical research.

## Methods

### Study design

The PRIDE Study is a national, online, longitudinal cohort study of the physical, mental, and social health of SGM people. The conception, development, implementation, and success of The PRIDE Study–Phase 1 in engaging and recruiting a diverse sample of SGM people are discussed here. The PRIDE Study–Phase 1 launched on June 25, 2015 to engage SGM communities in proposing and discussing health-related topics for research in The PRIDE Study and to create a snapshot of the cohort’s health. To do this, we designed and deployed an Apple iPhone app with the following goals: (i) recruiting a large diverse sample of SGM adults, (ii) gathering participant-level demographic and health data, and (iii) engaging the SGM communities in research question generation and prioritization. Data and lessons learned from Phase 1informed Phase 2, which launched via a web-based portal (pridestudy.org) accessible from any Internet-connected computer, tablet, or smartphone on May 2, 2017.

### Mobile app design and development

Apple announced the release of ResearchKit (researchkit.org), a framework to more easily develop iPhone apps for medical research, on March 9, 2015. Apple chose to facilitate the development of an iPhone app for The PRIDE Study as one of six initial apps released on the platform.

Because we did not have in-house software development expertise, we partnered with a technology design firm (THREAD Research) for app design and development. We were responsible for study concept, study design, and ensuring that app design and functionality met our study’s needs. THREAD Research was responsible for gathering and implementing the business and functional requirements that we desired in our iPhone app, designing a visually-appealing and engaging user interface/experience (UI/UX), coding the app employing the ResearchKit framework, and setting up the cloud-based backend (*i*.*e*., compute and database instances, storage) for proper data collection and storage. We had full access to the study data and conducted all analyses.

App development occurred rapidly over four weeks using the scrum software development framework.[28] The development team included one scrum master, one iOS developer, one back-end developer, one product designer, one digital producer, one quality assurance specialist, and two executives (Vice President of Digital, Chief Operating Officer). We had frequent (*i*.*e*., daily, every other day) “sync sessions” with the THREAD Research team to decide key design elements and ensure all questions relating to current and upcoming development were answered. Rigorous load testing, testing the app on different iPhone versions, software development quality assurance (QA) checks, bug remediation, user acceptance testing, and live QA review were performed by THREAD Research under our supervision.

### Data management and security

All data transmissions from app users to the cloud computing services (Amazon Web Services, AWS; Seattle, WA) were encrypted using 256-bit Secure Sockets Layer (SSL) cryptographic protocols. A secure load balancer forced encrypted connections and distributed the workload across several compute instances as needed. All app account data (except Community Forum data) were stored in AWS using services provided by Aptible (New York, NY). In this infrastructure, the database was within a private subnet (not accessible from the Internet) inside an AWS Virtual Private Cloud. All data were encrypted in transit and at rest. Identifying information (first name, last name, e-mail address, date-of-birth) was stored in a database separate from survey data. All community forum data were stored in a MongoDB (NoSQL) database running on Heroku (San Francisco, CA). Survey data (linked to the participant by participant ID number) were stored in Qualtrics Research Suite (Salt Lake City, UT) to facilitate easy reporting and analysis.

### Regulatory and compliance

Final versions were submitted to the Apple App Store for review under the University of California, San Francisco (UCSF)’s developer account. The first version of the app was available on June 25, 2015. The UCSF Institutional Review Board (IRB) approved this study (Study Numbers: 15–16697 and 16–18773).

In addition to required approval from the UCSF IRB, we engaged with other university offices to ensure compliance and mitigate any future risk. The UCSF Information Technology Security group required a security assessment to evaluate data protection procedures and breach risk. UCSF Risk Management, Counsel, and Privacy Office all reviewed and edited the app’s Privacy Policy (S1 File) to ensure that prospective participants were educated about how we collect, protect, and use collected data. UCSF Brand Identity ensured compliance with the university’s use of name, logo, typefaces, and colors. Additionally, UCSF Public Relations worked to secure earned media coverage of the app launch in accordance with university policies.

### PRIDEnet: Community engagement network

Prior to the development and launch of the app, a preliminary market survey was conducted by Salesforce to understand potential participant motivations for participating in an electronic health study of SGM people. We subsequently developed PRIDEnet, a participant-powered research network and community engagement mechanism designed to catalyze SGM health research by building relationships, recognizing multifaceted identities, creating equity among SGM communities, and maintaining transparency in our work. PRIDEnet (pridestudy.org/pridenet) is currently composed of a national participant advisory committee, ambassadors, and a national community partner consortium of approximately 41 SGM-focused non-profit health clinics, community centers, and advocacy/professional organizations to engage participants in research (including The PRIDE Study), publicize study launch broadly, and disseminate study results back to SGM community members. The community partner consortium members are active, known organizations within their local communities and serve as trusted partners to their constituencies to help ensure that SGM voices from across the country are influencing The PRIDE Study.

### Publicity and recruitment

**Fig 1:**
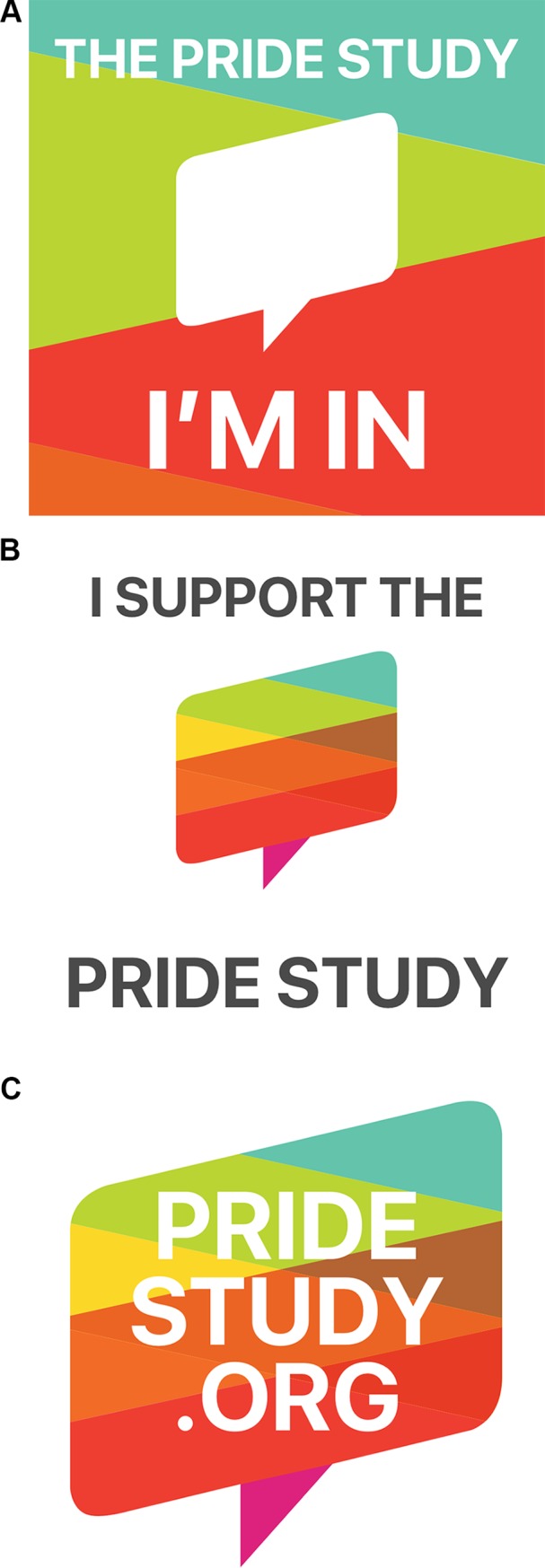
Social media sharing images for the PRIDE study. (A-C) Selected images used to promote The PRIDE Study on social media.

In preparation for launch, we created a variety of digital collateral media to ensure a uniform brand and digital presence including The PRIDE Study website (pridestudy.org) and a brand style guide (logo, font, colors). The website provided a single location for potential participants to learn about the research, potential benefits to the SGM community, the team conducting the research, the privacy policy, and how to obtain the app (or participate without an iPhone). Because of our focus on engaging the SGM community, we developed a video (available at https://youtu.be/ierbGIDFH_8) with SGM community members telling their health care experiences. We developed several social media sharing images (Fig 1A–1C) for people to share via Facebook and Twitter to promote the study. We set up a text message shortcode–text “PRIDESTUDY” to 74121 –to provide interested potential users with a direct link to download the app. We ran paid advertisements on Facebook and Twitter for two weeks after app launch. In addition to advertising, traditional media–including print articles in the *Washington Post* and a front-page story in the *San Francisco Chronicle*, online articles from Buzzfeed and UCSF, and a radio interview on National Public Radio–assisted with recruitment. We provided information about the study and the iPhone app to our PRIDEnet community partner consortium for distribution to their constituencies. Recruitment was thereby performed using convenience and snowball sampling after the numerous aforementioned publicity events.

### Eligibility screening and enrollment

**Fig 2:**
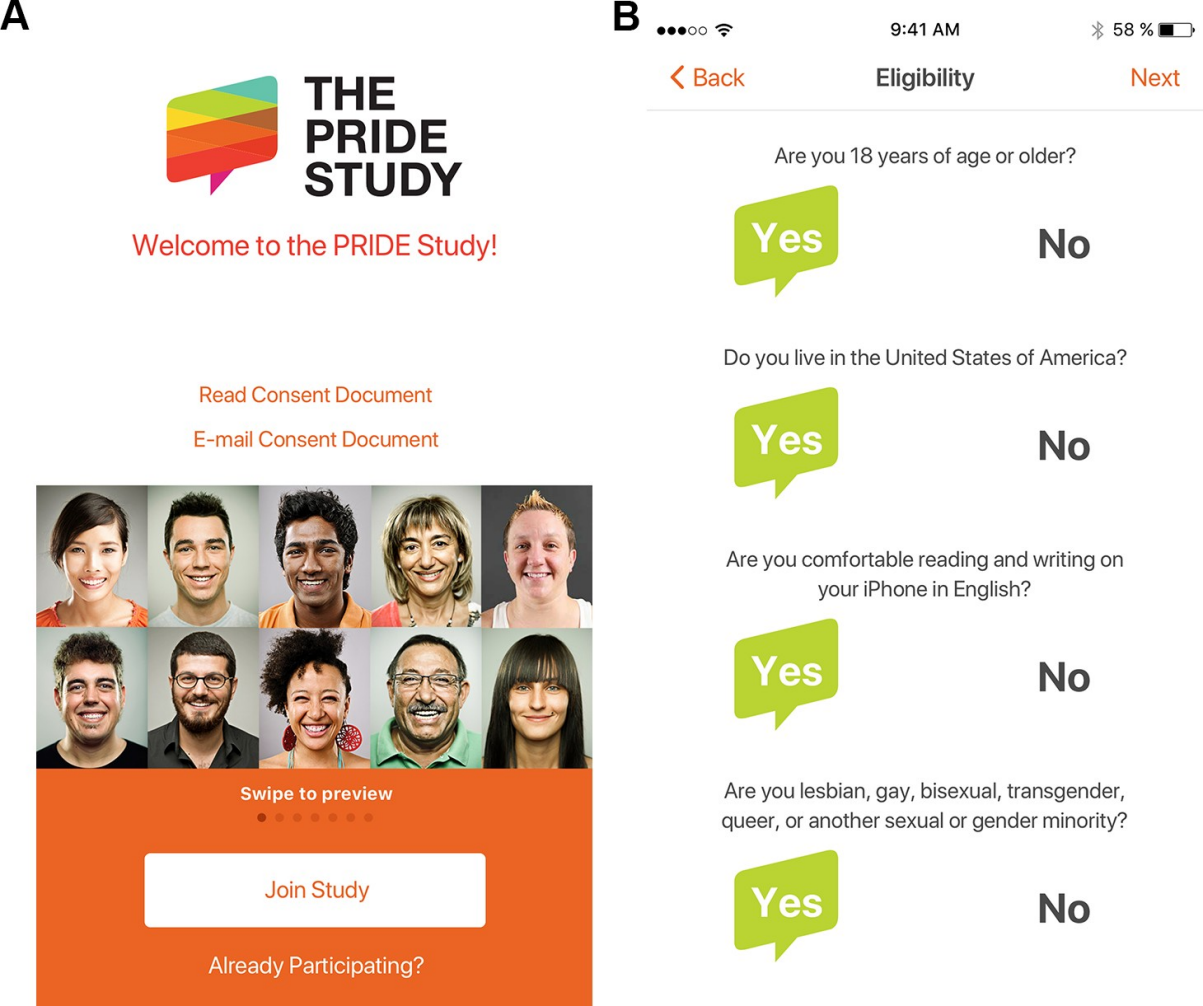
The PRIDE study iPhone app. (A) Welcome screen. (B) Eligibility screening questions.

**Fig 3:**
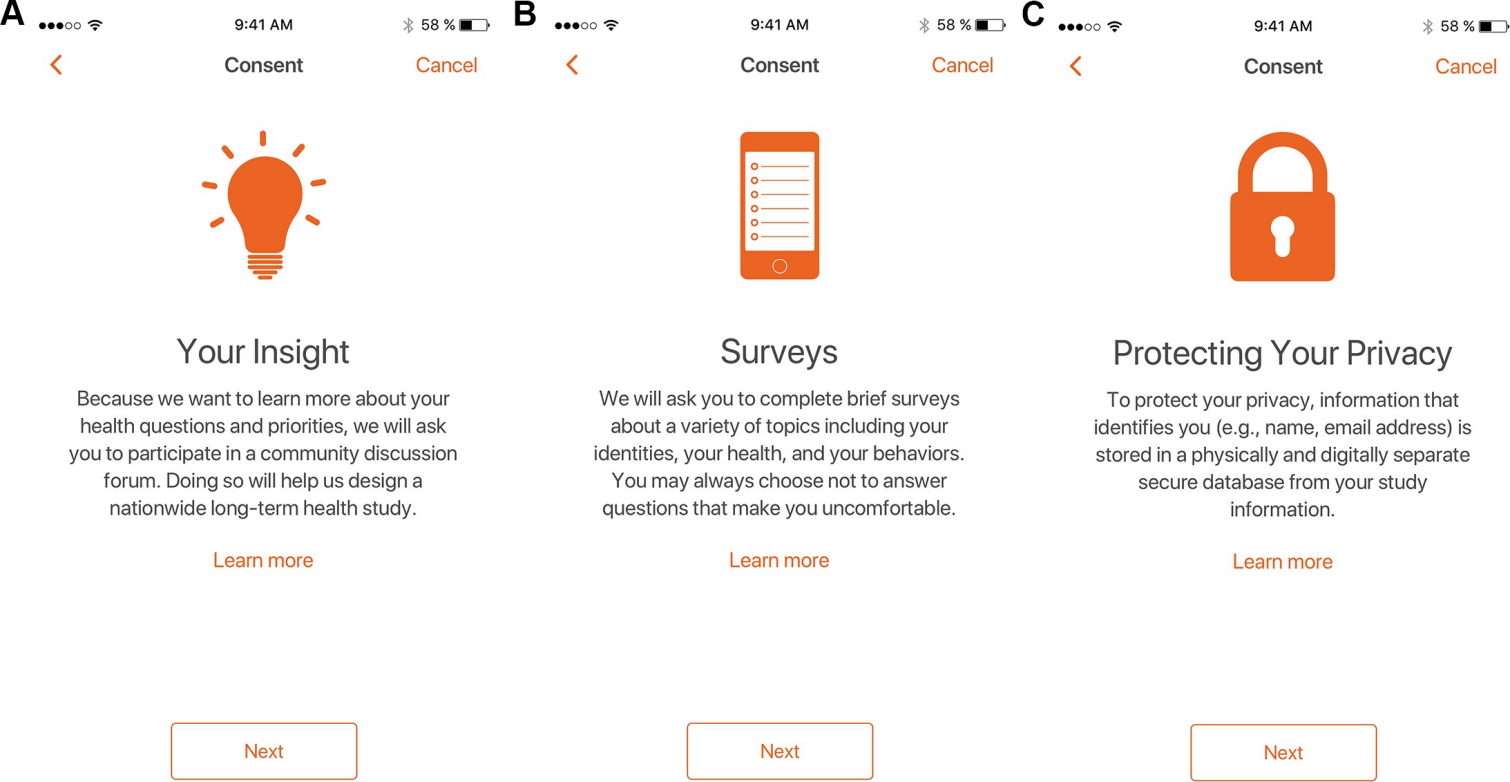
The PRIDE study iPhone app: Informed consent. (A-C) Example screens to guide participants step-by-step through informed consent.

Interested participants who downloaded and installed the app from the Apple App Store were presented with a welcome screen that provided study and consent information (Fig 2A). For those wishing to join the study, Boolean eligibility screening questions were presented (Fig 2B). People were eligible to participate in The PRIDE Study–Phase 1 if they (i) were at least 18 years of age, (ii) living in the United States, (iii) were a sexual and/or gender minority, (iv) were comfortable reading and writing in English, and (v) had access to an Apple iPhone. Eligible participants were subsequently directed through a series of screens (analogous to an in-person consent discussion) explaining study activities, time commitment, data security, potential benefits, etc. (selected examples in Fig 3A–3C). Participants were subsequently required to successfully pass a comprehension quiz about study participation before affirming their desire to participate and providing their name, date-of-birth, e-mail address, ZIP code, and digital signature. Enrollment was completed when participants verified their e-mail addresses by visiting a unique link sent by e-mail. Participants were also able to authorize sharing of Apple HealthKit data (*i*.*e*., height, weight, steps walked, flights climbed, distance traveled).

In addition to enrollment procedures, the app contained a community discussion forum, surveys, and a real-time cohort data dashboard (all discussed in detail below). In response to participant feedback, a new version of the app was released on September 11, 2015 that included design changes to better represent gender identity data from the transgender community, to add push notification functionality to notify participants of new study activities, and to provide bug fixes.

### Community forum

**Fig 4:**
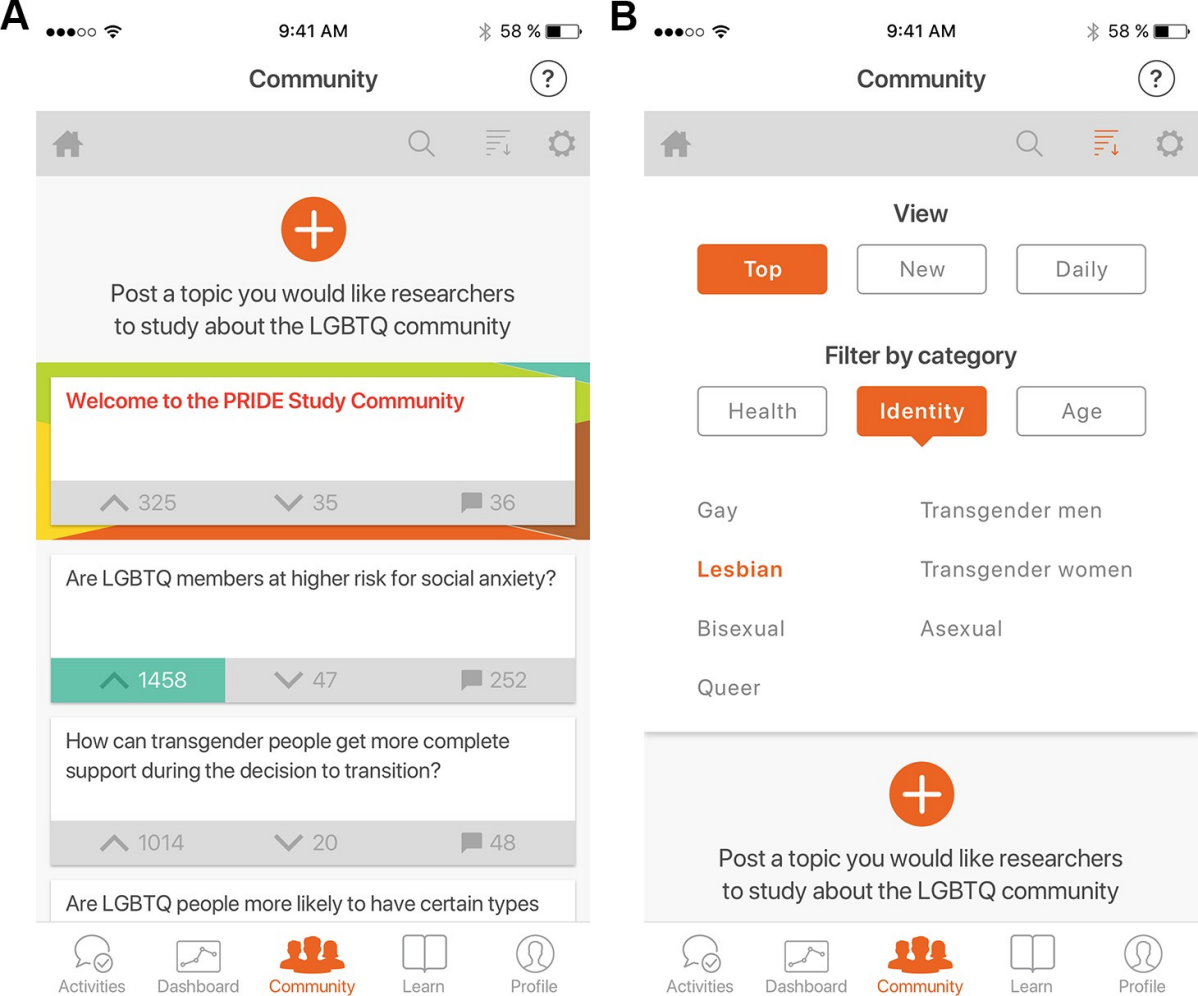
The PRIDE study iPhone app: Community forum. (A) Participants can quickly browse submitted research topics with associated numbers of upvotes, downvotes, and comments. (B) Participants submit a research topic, the reason it is important, and the appropriate categories.

To engage study participants in a discussion forum about SGM health-related topics, we developed a community module (using Telescope–telescopeapp.org–as this functionality did not exist in ResearchKit) (Fig 4A). Participants selected a screen name, which was not linked to their PRIDE Study iPhone app account or any survey responses. Participants were invited to “post a topic [they] would like researchers to study about the LGBTQ community” (Fig 4B) and classify the topic by health (mental/emotional, physical, social), identity (asexual, bisexual, gay, lesbian, queer, transgender men, transgender women), and age (children and teenagers– 0–18 years-old; young adults– 18–40 years-old; middle-aged adults– 41–65 years-old; senior adults– 65+ years-old). Participants could reply to topics posted by others to start a discussion thread and could vote each topic up (favorable) or down (unfavorable) to indicate their opinion of the topic. Participants could “follow” a conversation to be notified of new discussion and could “flag” conversations as inappropriate for investigation by study staff.

### Surveys

We developed five surveys: (i) Demographics, (ii) Physical Health, (iii) Mental Health, (iv) Social Health, and (v) Improving The PRIDE Study. The Demographics survey was released on June 25, 2015; all other surveys were released one year later on July 25, 2016. With the exception of items assessing sexual orientation and gender identity, existing survey items from federal studies (*e*.*g*., National Health Interview Survey) and validated scales were used when available. For sexual orientation and gender identity items, we combined items created by several groups [29–31] in order to have more comprehensive answer choices (Obedin-Maliver *et al*., under review). Surveys were piloted and subsequently modified to improve understanding, usability, and burden.

## Results

### Participant recruitment and demographics

**Fig 5:**
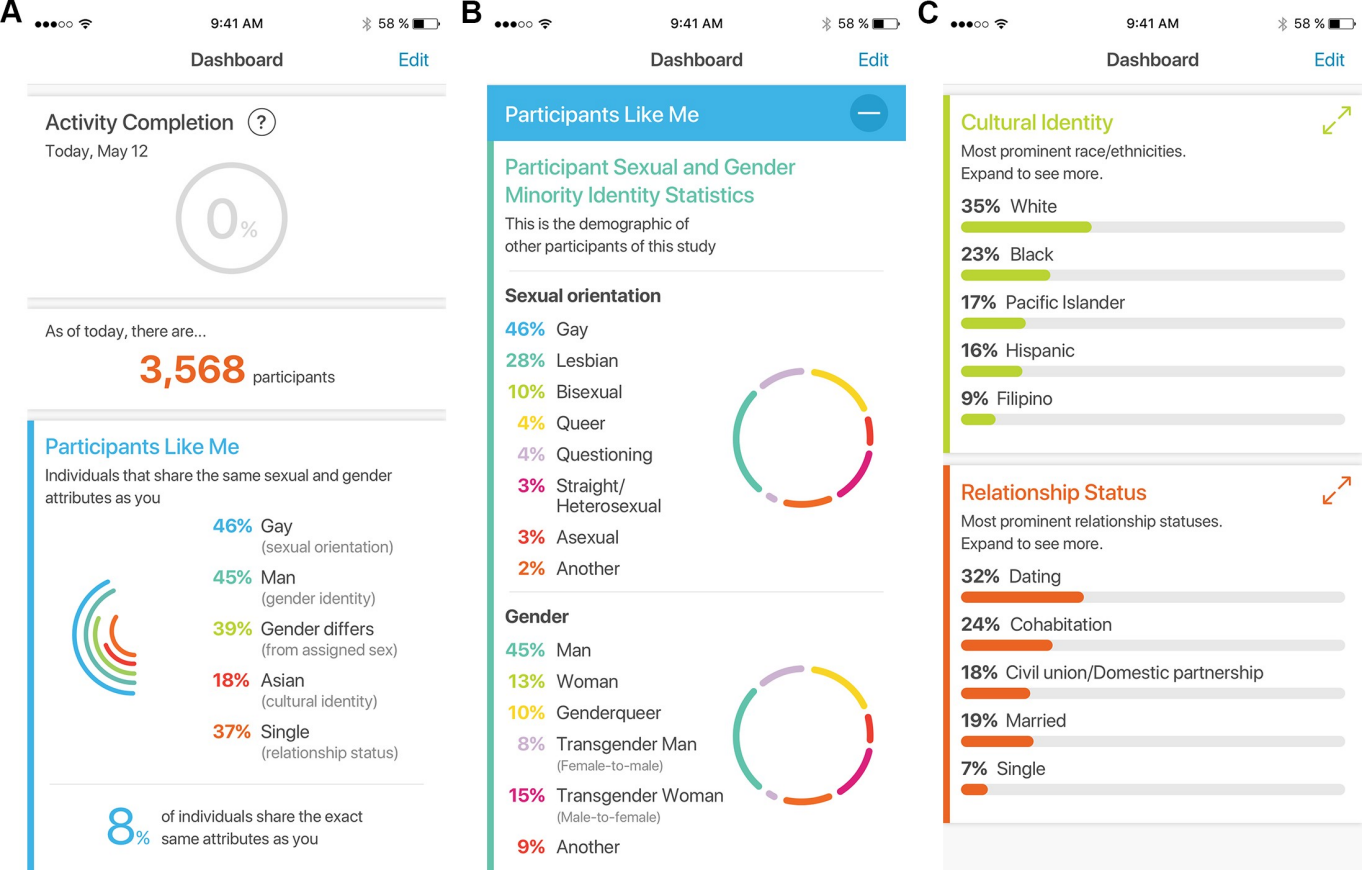
The PRIDE study iPhone app: Real-time dashboards. (A) Overall enrolled participant count and distribution of participants with similar demographics. (B) Distribution of participants’ sexual orientations and gender identities. (C) Distribution of participants’ racial/ethnic identities and relationship statuses.

During this phase, The PRIDE Study iPhone app was downloaded 35,168 times with 18,099 participants completing consent and 16,394 participants providing data (*i*.*e*., completing any survey). Of the 16,385 participants providing demographic data, more than 98% identified as a sexual minority (*i*.*e*., identified as anything but exclusively heterosexual), and more than 15% identified as a gender minority (*i*.*e*., gender identity that is not congruent with sex assigned at birth). The median age was 27.6 years (IQR, 22.6–36.5). Complete sociodemographics are available in Table 1. Participants reported significant digital connectedness with 95.9% using social media at least once per week. Participants each had an average of 3.15 social media accounts (standard deviation, 1.70). Participants self-reported numerous “other” sexual orientations and gender identities not captured by our provided answer choices; we are reporting these details elsewhere (manuscript in preparation). Participants were able to view real-time cohort statistics of sexual orientation, gender identity, race/ethnicity, and relationship status via a participant dashboard (Fig 5A–5C).

**Table 1:** Participant sociodemographics.

Characteristic	N (%)
Gender Identity (N = 16,329)	
Genderqueer	621 (3.8%)
Man	8,333 (51.0%)
Transgender man	286 (1.8%)
Transgender woman	204 (1.3%)
Woman	5,501 (33.7%)
Another gender identity	420 (2.6%)
More than 1 gender identity	964 (5.9%)
Sex Assigned at Birth (N = 16,316)	
Female	7,323 (44.9%)
Male	8,993 (55.1%)
Sexual Orientation (N = 16,342)	
Asexual	289 (1.8%)
Bisexual	2,244 (13.7%)
Gay	7,191 (44.0%)
Lesbian	2,111 (12.9%)
Queer	755 (4.6%)
Questioning	155 (1.0%)
Straight	166 (1/0%)
Another sexual orientation	702 (4.3%)
More than 1 sexual orientation	2,734 (16.7%)
Gender Minority (N = 16,316)	2,515 (15.4%)
Sexual Minority (N = 16,316)	16,026 (98.2%)
Sexual and Gender Minority (N = 16,316)	2,387 (14.6%)
Race / Ethnicity (N = 16,174)	
African-American	568 (3.5%)
American Indian or Alaska Native	66 (0.4%)
Asian/Pacific Islander	660 (4.1%)
White	12,946 (80.0%)
Mixed race	1451 (9.0%)
Another race	483 (3.0%)
Hispanic/Latino/Spanish Ethnicity (N = 16,300)	2,189 (13.4%)
Born in the U.S. (N = 16,324)	15,132 (92.7%)
Education (N = 16,078)	
Less than high school	401 (2.5%)
High school graduate or equivalent	2,958 (18.4%)
Trade/Technical/Vocational training	307 (1.9%)
Some college	4,566 (28.4%)
2-year degree	894 (5.6%)
4-year college degree	4,311 (26.8%)
Graduate degree (Masters/Doctoral/Professional)	2,641 (16.4%)
Income (Annual Individual) (N = 14,813)	
$0–10,000	4,250 (28.7%)
$10,001–20,000	1,999 (13.5%)
$20,001–40,000	2,518 (17.0%)
$40,001–60,000	2,141 (14.5%)
$60,001–80,000	1,180 (8.0%)
$80,001–100,000	702 (4.7%)
>$100,000	1,573 (10.6%)
Ever Served in U.S. Armed Services (N = 16,301)	571 (3.5%)
Have Health Insurance (N = 16,061)	14,797 (92.1%)
Relationship Status (N = 16,275)	
Married	1,870 (11.5%)
Civil Union/Domestic Partnership	392 (2.4%)
Live with Partner (cohabitation; no legal document)	2,786 (17.1%)
Dating, not living together	3,287 (20.2%)
Separated/Divorced/Widowed	366 (2.2%)
Single; Never Married or Civil Union/Domestic Partnership	7,368 (45.3%)
Another relationship status	206 (1.3%)
Region (N = 2,250)^1^	
Northeast	429 (19.1%)
Midwest	382 (17.1%)
South	658 (29.2%)
West	779 (34.6%)

^1^ Region determined by participant-entered ZIP code among participants who completed the Physical Health, Mental Health, or Social Health surveys.

### Participant engagement and experience

Nearly all consented participants (n = 16,385; 99.95%) completed the Demographics survey while only a relatively small minority completed the Physical Health (n = 2,075; 12.7%), Mental Health (n = 1,833; 11.2%), Social Health (n = 1,803; 11.0%), and Improving The PRIDE Study (n = 1,926; 11.8%) surveys. The median completion time for each survey was less than ten minutes: 7 minutes 20 seconds for Physical Health, 5 minutes 11 seconds for Mental Health, and 8 minutes 55 seconds for Social Health, and 2 minutes 25 seconds for Improving The PRIDE Study. Participants reported that they would be willing to complete an average of 7.89 (standard deviation 4.25) 30-minute surveys annually. Participants were asked “what will keep you coming back and completing surveys annually for The PRIDE Study?” and most frequently selected: updates about study results (59.7%), feedback about how my participation is helping the study (51.7%), and incentives (*e*.*g*., gift cards, giveaways; 31.7%). More than one-quarter (25.6%) selected “I don’t need anything in return. I will keep participating to improve LGBTQ health.” Physical activity information (collected by iPhone sensors) was shared by 5270 (32.2%) participants.

In the community forum module, 3,544 health topics were provided by 3,089 participants (average 1.14 health topics posted per user, range 1–8). There were 5,063 responses from 3,174 participants (average 1.6 responses per user, range 1–38) posted about these health topics. There were 56,341 up votes and 4,181 down votes cast by participants to indicate their opinion of a particular topic. Fewer than ten comments required moderation by the study staff for inappropriate/offensive language including anti-SGM language. The “Top Five” discussion topics (algorithm based on number of views and number of up votes) were presented in the live participant-facing dashboard, along with number of topics posted daily, and a breakdown by health, identity, and age classifications. Participant-proposed SGM health research topics and health survey data will be presented elsewhere.

### App development and study costs

Our estimated costs were approximately $605,000 including ~$300,000 for researcher/staff salaries (two fellows at $75,000 per person per year for two years), ~$250,000 in app development costs (provided *pro bono* by THREAD Research), ~$45,000 in cloud computing expenses (provided *pro bono* by Aptible and THREAD Research), and ~$10,000 in advertising. We did not separate participant recruitment from other study costs; our costs included app design and development, cloud computing and database services, recruitment of 18,099 participants, completion of 24,022 surveys, and robust community forum engagement with 3,544 posted health topics, more than 5,000 comments, and more than 60,500 votes cast by participants. With 18,099 consented participants, this equates to $33.43 per participant inclusive of all study infrastructure and activities.

## Discussion

The PRIDE Study–Phase 1 utilized an innovative iPhone app that successfully recruited a diverse national sample of nearly 20,000 SGM people who completed over 24,000 surveys and provided more than 3,500 health topics for future study in The PRIDE Study–Phase 2. This is believed to be the first time that an app has specifically engaged and recruited large numbers of an underrepresented and understudied population for clinical research [27]. It proved to be a cost-effective way to engage participants in providing and prioritizing health-related research questions/concerns and completing surveys. Similar approaches may be effective in other populations that are also underserved, understudied, and vulnerable to poor health outcomes. With stigmatizing, discriminatory, or dangerous experiences in society, health care, or investigational communities, potential participant interactions with the health care system and traditional research enterprise may be limited out of fear. The online approach, however, avoids in-person interactions and may thereby make research study participation safer and more comfortable by enabling high quality control for omitting stigmatizing content that may arise during interpersonal interactions [7–10].

With the development of mHealth technologies including consumer health devices (*e*.*g*., activity trackers) [32] and increasing Internet-connected device ownership [33], clinical research is changing. Clinical research is moving away from the traditional in-person model that recruits participants to come to medical centers for research or recruits patients into research studies through their interactions with the health care system. Such studies are generally inconvenient (due to travel, parking, scheduling, etc.), and particularly so for participants with limited mobility, who live far from academic medical centers, and who are unable to take time off from work. Despite significant development costs, digital research is appealing to researchers due to the ability to reach a large geographic region, more facile recruitment (in part due to less invasive/intense study designs), and decreasing per-participant costs as additional participants enroll. These advanced technologies enable clinical research study recruitment and participation (*i*.*e*., data collection) on a more frequent basis from various sources (*e*.*g*., surveys, consumer health devices, smartphone location and sensor data) in participants’ natural environments (*e*.*g*., home, workplace) without the burden created by in-person visits. These benefits are balanced with significant concerns, especially in today’s sociopolitical climate, about data security and privacy. Methods (and appropriate messaging to participants) to increase data security (*e*.*g*., two-factor authentication, encrypting databases at rest and in transit, limiting administrator access to a certain IP address range, etc.) and ensure participant privacy (*e*.*g*., establishing methods to deidentify data, data use agreements to ensure data is released to researcher committed to improving SGM health) are critical to building trustworthiness.

Like The PRIDE Study, several digital cohort studies have successfully recruited high numbers of participants in recent years. Stanford’s MyHeart Counts Study (a ResearchKit-powered iPhone app) recruited nearly 50,000 participants, collected survey-based participant-reported data including laboratory values, and utilized the iPhone sensors to conduct 6-minute walk tests [17]. The Health eHeart Study (health-eheartstudy.org) is a web-based longitudinal cohort study based at the University of California, San Francisco that has recruited over 180,000 consented participants [34]. Other studies have utilized mobile technologies to study disease in new ways: image-based analysis of moles to study melanoma [20], geofencing to assess hospitalizations [35], and geolocation in combination with ecological momentary assessments to assess asthma triggers [18].

Community engagement improves trust and willingness to participate in research by engaging stakeholders in creating and maintaining patient/participant-centered approaches [36]. Technological advances have expanded community engagement techniques by moving traditional in-person experiences to digital spaces. Community engagement strategies, such as online focus groups or crowdsourcing of research questions (as we did here), have enabled researchers to harness the power of large numbers in generating data, analyzing data, or generating novel research questions [37, 38]. In longitudinal digital studies where participants do not form in-person relationships with study staff, community engagement techniques to foster ownership of and belonging in research studies may be particularly important to retain active participation. Finally, returning research results directly to participants demonstrates the immediate outputs of individual participation by returning the data (and interpretation of those data) for a specific population. While ethical, legal, and regulatory issues abound, the 2018 revisions to the Federal Policy for the Protection of Human Subjects (“the Common Rule”) signal this evolution to a more participatory biomedical research model [39].

Our digital approach has many strengths. First, a large, diverse, national sample of SGM people was successfully recruited with participants from many age, racial, ethnic, educational, and socioeconomic groups. Second, the average cost-per-participant was significantly less than typical observational clinical research studies. Pharmaceutical clinical studies, for example, ranged in cost from $1.4 to $52.9 million depending on study procedures, therapeutic area, and phase [40]. Third, participation was low burden, accessible to anyone with access to an iPhone, and occurred in participants’ natural environments without requiring clinical research physical or human resources. Fourth, participants exhibited high engagement by completing surveys and readily providing their current health concerns and commenting and voting on concerns posted by others. Fifth, research data were returned to participants via a real-time dashboard. This also permits researchers to easily track the cohort composition and alter recruitment efforts as needed. Sixth, passive collection of activity-based data was successful using Apple’s HealthKit (developer.apple.com/healthkit/) code framework.

Our work has several limitations. First, we recruited a convenience (non-probability) sample. This limitation is inherent in studies involving communities with limited population-level data (*e*.*g*., SGM people) to generate probability sampling frames. Sexual orientation and gender identity have not been previously collected and will not be collected by the US Census Bureau for the 2020 Census or American Community Survey [5], which are used as the sampling frames for other large scale studies [4]. Comparing our sample to U.S. Census data is not possible. Despite this limitation, important within-sample comparisons remain possible. Second, our sample was not as racially/ethnically diverse as we would have liked. This may be due to the demographics of Apple iPhone ownership, online advertising that did not target specific populations, the audience demographics of our earned media coverage, etc. Specific community engagement attention to diverse subcommunities may recruit and retain a more heterogeneous sample. Third, our app-based approach without an in-person component limited the data types that can be collected or verified, and it limited participation to iPhone owners. Fourth, the low response rate to some surveys was likely due to a long lag between initial launch and deployment of the physical health, mental health, and social health surveys; a shorter data collection period for these surveys; and limited notifications to participants about the new surveys. Continued engagement with surveys or other activities may be an important aspect for continued participation in digital longitudinal cohort studies. Fifth, we did not verify participant identity. As such, our sample could be contaminated by duplicate participants and non-SGM-identified people (*e*.*g*., those who might be curious about the study or press). As no monetary incentive was offered for participation, the impetus for multiple accounts created by a single user was low. Finally, we did not collect app engagement metrics (*e*.*g*., daily app opens, daily active users, daily sessions, churn rate, etc.) to measure user engagement with our iPhone app.

We encountered and learned from several technical challenges. First, incomplete bug and quality assurance testing resulted in the date-of-birth of many participants not being stored in the database upon enrollment. We were able to obtain a significant fraction of these missing data via an updated app and by asking participant date-of-birth in other surveys. Second, the high volume of responses in the community forum resulted in slow loading. This, along with limited functionality to sort and filter data, frustrated participants and limited their ability to participate fully. We suspect that our engagement would be even higher with a higher performance, user experience-optimized community forum. Third, at the time of our ResearchKit app release, the early ResearchKit framework had several limitations including cumbersome coding for survey branching, the inability to push surveys live directly to participants (downloading an app update is required), and the inability to link results of one survey to another. (The ResearchKit framework has since been expanded, and ResearchStack (researchstack.org)–a similar software development framework for Android–has been developed.)

The approach that we described here may be adapted for other communities/populations. Partnering with engaged community members as meaningful, valued stakeholders in the study design and app development processes can help vet decisions while simultaneously building a group of trusted partners. Adaption to another population requires updating the language, imagery, and study procedures (*e*.*g*., survey questions) to resonate with the communities of interest; inclusive, culturally competent/humble studies enable potential participants to see themselves as welcomed participants. Depending on the historical or current transgressions, additional work may be needed to combat mistrust that many populations have of the medical or investigational communities. Engaging community members throughout the entire process creates a study that was truly designed *with* the community, rather than *for* the community. Digital research platforms–like the new NIH-funded Eureka Research Platform (info.eurekaplatform.org)–enable researchers to create mobile health research studies customized to the specific study without the need to set-up expensive computing infrastructure.

We chose to not pursue further development of our iPhone app for several reasons. First, the high cost of iPhone devices (and the lack of pre-paid iPhones) limited the diversity of the SGM communities that we could reach. Second, as noted above, deploying additional surveys could not be pushed live directly to participants in the initial ResearchKit release (*i*.*e*., participants had to download an app update to get new surveys). Third, including Android users would have required us to conduct parallel app design and development of an Android app. All app updates would also need to be done on both platforms. Non-iPhone/Android smartphone users would still be unable to participate.

We decided to retire our iPhone app and develop a web-based research portal accessible from any Internet-connected device (*i*.*e*., computers, tablets, all smartphones) to allow for broader accessibility of diverse populations including those who do not own a smartphone and to enable immediate deployment of new surveys to participants without requiring additional steps by the participant. This digital research platform features automated eligibility screening, consenting, and enrollment along with state-of-the-art data security and numerous integrations with services that permit facile survey design and administration, cohort segmentation, and e-mail- and text message-based notifications. Enrolled participants complete health profiles, annual health questionnaires, quarterly hospitalization assessments, and topic-specific cross-sectional ancillary studies (*e*.*g*., breast cancer screening study distributed only to those assigned female sex at birth). In addition to participant-generated health data, mailing addresses are automatically geocoded to provide additional details about participant’s environments (*e*.*g*., Census tract), and participants can link a variety of mHealth devices including activity trackers, smart scales, and blood pressure cuffs. We are reporting details about our custom web-based research portal elsewhere (manuscript under review).

In conclusion, we developed an iPhone app that enabled the recruitment of an underserved and understudied population into a national online cohort study of SGM adults. Existing software frameworks facilitated app development and the collection of data from a large, diverse national sample at lower cost than traditional cohort studies. Digital engagement features (*e*.*g*., Community Forum) empowered participants to become engaged stakeholders in the research development process and provided them with real-time data via visually appealing dashboards to help maintain engagement. Mobile app-based approaches may be successful, convenient, and cost-effective at engaging and recruiting other vulnerable populations into clinical research studies.

## Supporting information

S1 File The PRIDE study iPhone app: Privacy policy. (PDF)
