# Supplementary material for: Using mobile technology to engage sexual and gender minorities in clinical research
Source: PLoS One. 2019 May 2;14(5):e0216282. doi: 10.1371/journal.pone.0216282 (PMC6497300; doi:10.1371/journal.pone.0216282)
Supplement: S1 File — (PDF) [file pone.0216282.s001.pdf]

The PRIDE Study iPhone App: Privacy Policy

The Regents of the University of California, San Francisco (“UCSF”) and The PRIDE (Population Research in Identity and Disparities for Equality) Study (collectively known as the “Research Team”) know that you care about how your information is used and shared. The Research Team takes protecting your privacy very seriously. Please read the following to learn more about how the Research Team uses and protects information collected through The PRIDE Study app (the “App”).

By using the App in any manner, you acknowledge that you accept the practices and policies outlined in The PRIDE Study App Privacy Policy (this “Privacy Policy”).

You consent that the Research Team will collect, use, and share your information as described in this Privacy Policy.

What does this Privacy Policy cover?
This Privacy Policy covers the treatment of personally identifiable information ("Personal Information") that the Research Team gathers when you are accessing or using the App. This Privacy Policy does not apply to the practices of companies or institutions that the Research Team does not own or control, or who are not members of the Research Team.

Your use of the App will allow you to provide the Research Team with Personal Information, which may include but is not limited to information about your identity and other demographic information and your physical activity. The App collects your Personal Information for the purposes of a research study being conducted at UCSF called the “The PRIDE (Population Research in Identity and Disparities for Equality) Study” (the “Study”). **If you choose to use the App to provide the Research Team with any Personal Information, you must first review and acknowledge the informed consent form for the Study, which will be presented to you within the App.**

The Research Team does not knowingly collect or solicit Personal Information from anyone under the age of 18 or knowingly allow such persons to register for The PRIDE Study App. If you are under 18, you must not register for the App. In the event that the Research Team learns that the Research Team has collected Personal Information from a person under age 18, the Research Team will delete that information. If you believe that the Research Team might have any information from or about a person under the age of 18, please contact the Research Team at pridestudy@ucsf.edu or 855-421-9991 (toll-free).

How does The PRIDE Study App collect information?
The App will obtain information from you in two ways. The first is information that you manually enter into the App. The second is information that the App collects automatically about you.

Information you manually enter into the App:
The Research Team receives and stores information you manually enter into the App. For example, the Research Team collects Personal Information that directly identifies you, including

your name, email address, and birth date. The Research Team collect this information to verify (i) your identity, (ii) that you reside within the United States, (iii) that you are over 18 years of age, and (iv) so that the Research Team can contact you. In addition to these direct identifiers, the Research Team asks you to enter information including gender identity, sexual orientation, education level, weight, height, annual income, race, ethnicity, health insurance status, United States Armed Services affiliation, etc. Additionally, the Research Team may ask about your health care experiences. You can choose not to provide the Research Team with certain information.

Information collected automatically:
In addition to the Personal Information that you manually enter, the App also collects certain Personal Information about you automatically through your smartphone and/or any other wearable device which you use for the Study. For example, if you have affirmed your consent to do so, the App will interface with your smartphone or wearable device's sensors (accelerometers, gyroscope, barometer, etc.) to collect your physical activity. These data will be automatically uploaded to your account with us when you use the App.

The App may also collect information about your location, including real-time location, through your smartphone or wearable device's applicable geolocation, GPS, Wi-Fi or similar capabilities. The Research Team will use the location information for the purpose of tracking your physical activities as well as locations that you visit (*e.g.*, hospitals).

This App may also automatically receive technical information relating to your usage of the App such as your operating system, device, features used, content viewed and downloaded, the dates and times of your interactions with the App and other information. The Research Team may use this information to understand, customize, and improve user experience with the App and the Study.

Push Notifications
"Push notifications" are messages that are sent to your smartphone to remind you of what you need to do in the Study. The App may send you push notifications throughout your participation in the Study.

Is Personal Information about me secure?
All information that is collected through the App will be sent to a secure data server run by Aptible. The PRIDE Study currently utilizes 128-bit secure socket layer (SSL) protocol which protects all data transmission sent between our servers, Aptible, and the App. Direct identifiers will be replaced with a code to help protect your identity. Aptible's security practices have been reviewed to meet privacy and security standards set by UCSF.

The direct identifiers will be encrypted and stored separately from your study data. UCSF researchers will, however, maintain your consent and personal information and retain the ability to re-identify the information for research or legal purposes, and they may share re-identified information with others at UCSF who need to see such information to ensure that the research meets legal, regulatory, or institutional requirements.

Access to the App on your smartphone is protected by TouchID and/or the passcode you selected after you downloaded the App. One of these needs to be entered every time you access the App. You should take steps to prevent unauthorized access to your account and personal information by selecting and protecting your TouchID and passcode appropriately and limiting access to your smartphone.

The Research Team endeavors to protect the privacy of the Personal Information that the Research Team holds in its records, but **cannot guarantee complete security. Unauthorized entry or use, hardware or software failure, and other factors, may compromise the security of your Personal Information at any time.**

The App may contain links to educational websites. The Research Team is not responsible for the privacy policies and/or practices on websites that may be accessed through these links. When following a link to another website, you should read that website's privacy policy and make sure that you agree with it and can abide by it.

Please note that all information collected by the App will be processed in the United States, and this may be done by another entity under contract with UCSF. During the study, only UCSF and contractors engaged by UCSF will be able to compile data you generate or provide through or with the App. You should only transmit data for the Study if you are in the United States at the time you transmit such data. If you transmit data for the Study while you are outside of the United States (which you should not do), then laws in the United States may not protect your privacy to the same extent as the laws in the country from which you have transmitted data to the App.

Will UCSF share any of the information it receives?
As part of your participation in the study, and per your informed consent form for the Study, the Research Team may share your coded study data (data without identifiers) with researchers outside of UCSF under criteria established by UCSF.

The information collected by the App will not be shared with or sold for advertising purposes.

The Research Team reserves the right to disclose information collected through the App as required by law, when the Research Team believes disclosure is necessary to comply with a regulatory requirement, judicial proceeding, court order, or legal process served on us, or to protect the safety, rights, or property of UCSF or the public.

What Personal Information can I access?
Through the App, you may access, and in some cases, edit or delete the following information you've provided to us:

- name and password
- email address
- user profile information, including your height, weight, and demographic information

When you update or delete information, however, the Research Team may maintain a copy of the unrevised information in our records to track the data entry into the Study and to assure the integrity of the Study. The information you can view and update may change as the

App undergoes further development. Please note that any information that is automatically uploaded from the App (*e.g.*, geolocation information) cannot later be changed or updated. If you have any questions about viewing or updating information that the Research Team has on file about you, please contact us at pridestudy@ucsf.edu or 855-421-9991 (toll-free).

What choices do I have?
You may choose not to install the App. If you choose not to install the App, you will be unable to participate in the Study.

You may stop all collection of information by the App at any time by withdrawing from the study on the "Profile" page within the App or uninstalling the App. As explained further in the informed consent form, you will no longer be able to participate in the Study if you choose to uninstall the App. If you uninstall the App, the Research Team will retain all of information about you that was collected prior to that point and continue to use such information as described in this Privacy Policy. If you have questions about withdrawing, please contact us at pridestudy@ucsf.edu or 855-421-9991 (toll-free).

You may be able to add, update, or delete certain information as explained above. When you update or delete information, the Research Team may maintain a copy of the unrevised information in our records.

Re-Contact
The Research Team may use your Personal Information to contact you to ask if you would be willing to participate in additional research opportunities, including linking your App data with other health data, with your permission.

What limitations are there on my use of the App?
When you download the App, you receive a non-exclusive, non-transferable, non-assignable license (without the right to sublicense) to install and use the App solely for your personal, non-commercial use in connection with the Study. You must own or control the device on which you download the App. By downloading and using the App, you agree that you will not do anything to interfere with or disrupt the operation of the App, will provide only accurate and current information through the App, and will not impersonate anyone else in your use of the App. You further agree not to transmit content that you do not have the right to transmit or that infringes the rights of any party, and you agree to use the App in compliance with all applicable laws. You understand that the App or portions of it may be subject to patent, copyright, trademark and other intellectual property protection and that the ownership of software and other intellectual property related to the App, as well the goodwill associated therewith, remains with UCSF or its licensors. You agree that any improvements or other changes to the App or the Study itself are the sole property of UCSF.

Effective Date and Changes to this Privacy Policy
This Privacy Policy is effective as of June 25, 2015. The Research Team may amend this Privacy Policy from time to time. New versions of this Privacy Policy are effective when they are posted to our website at the following link: www.pridestudy.org/app/privacy.html. Unless stated in an updated version, use of information that the Research Team collects will be subject to the version

of the Privacy Policy in effect at the time such information is collected.

How is my Health app data handled?
You have the option to share data from your mobile device's iOS Health app with the App. Once you agree to share your data through and with the App, such data become covered by this Privacy Policy.

The App was developed using Apple's HealthKit and ResearchKit frameworks and thus complies with Apple's development guidelines. To view these guidelines, please visit developer.apple.com/app-store/review/guidelines/#healthkit.

Questions or concerns?
If you have any questions or concerns regarding the topics addressed in this privacy policy, please contact us at pridestudy@ucsf.edu or 855-421-9991 (toll-free).

Disclaimer
You understand that nothing accessed through this App constitutes professional medical advice. UCSF disclaims any representation or warranty, expressed or implied, concerning the accuracy or completeness of the information contained on the App. In no event shall UCSF be liable for any special, indirect or consequential damages or damages of any kind whatsoever, resulting from or arising out of or in connection with the use of this App.

UCSF and The University of California, San Francisco are trademarks of The Regents of the University of California, San Francisco or its licensors.

This Privacy Policy shall be governed by and construed in accordance with the laws of the State of California.
